# Shot peening increases resistance to cyclic fatigue fracture of endodontic files

**DOI:** 10.1038/s41598-021-92382-x

**Published:** 2021-06-21

**Authors:** Javier Nino-Barrera, Jose Sanchez-Aleman, Manuel Acosta-Humanez, Luis Gamboa-Martinez, Carlos Cortes-Rodriguez

**Affiliations:** 1 Faculty of Dentistry, Endodontics Program, Universidad Nacional de Colombia, Building 210, Office 301, Bogotá, Colombia; 2 Department of Physics, Universidad Nacional de Colombia, Bogotá, Colombia; 3 School of Dentistry, Endodontics Program, Universidad El Bosque, Bogotá, Colombia; 4 Department of Mechanic and Mechatronics, Universidad Nacional de Colombia, Bogotá, Colombia

**Keywords:** Health care, Materials science

## Abstract

The objective of this study was to assess the resistance to fatigue fracture of conventional nickel–titanium files after undergoing shot peening. Forty NITIFLEX endodontic files, number 30, were divided into two groups; one was submitted to shot peening treatment and the other was not. All instruments were tested for fatigue fracture in simulated canals with a TRI-AUTO ZX endodontic motor. One file of each group was subjected to a residual stress analysis by XRD. Finally, the fractured surface was observed and elemental analysis performed by means of SEM and EDX. Roughness analysis was made by focal variation microscope. The shot peening group showed greater resistance to fatigue fracture; there was no difference in the length of the fractured fragments. XRD results showed the presence of residual compression stresses in the file submitted to shot peening, a decrease in the interplanar spacing, and an increase in the full-width-at-half-maximum and the microstrains. SEM and EDX showed a ductile fracture with zones of fatigue and an equiatomic ratio between the nickel and titanium. Surface roughness increased after the file was subjected to the shot peening procedure. In conclusion, shot peening increases the resistance to fatigue fracture due to the presence of residual compression stresses in files manufactured from a conventional nickel–titanium alloy.

## Introduction

In the cleaning and shaping of the root canal, nickel–titanium (NiTi) files allow endodontic treatments to be performed that preserve the original root morphology, due to the superelasticity of the instruments manufactured with this alloy. Unfortunately, NiTi instruments can fracture inside the root canal during endodontic treatment, becoming a physical block that prevents disinfection beyond the point where the fractured fragment is located^1^. The fracture of endodontic files has been reported at a frequency between 0.7 and 7.4%^1^ and has also been mentioned as one of the intraoperative complications of endodontic treatment that significantly decreases its prognosis^2^.

There are three modes by which endodontic files can be fractured: torsional fracture, where a part of the file is locked inside the canal but nevertheless continues rotating, causing fracture of the file; fatigue fracture, where the file rotates inside the root canal and has alternating sectors of continuously changing tension and compression, depending on the curvature, which can initiate cracks, leading to fracture of the instrument; and finally a combination of the two aforementioned modes^3,4^.

Fatigue fracture has been reported as the most frequent type of fracture of endodontic rotary files, especially in root canals with acute curvatures^4–6^. Nowadays, the appearance of reciprocating instruments, such as WAVEONE (Dentsply Maillefer, Ballaigues, Switzerland) and RECIPROC (VDW, Munich, Germany), as well as the use of files such as PATHFILE (Dentsply Maillefer, Ballaigues, Switzerland) or PROGLIDER (Dentsply Maillefer, Ballaigues, Switzerland) for the glide path, have decreased the percentage of torsional fractures^7,8^. Despite the above, fatigue fracture has been prevented with measures such as a decreased number of uses^5,9^ and conventional NiTi alloy heat treatments^10^, which change the original characteristics of the alloy, making it more ductile and consequently reducing its superelasticity. Surface treatments such as electropolishing have also been proposed^11^ to try to prevent the origin or growth of cracks by making their surface smooth; however, their effectiveness in increasing resistance to fatigue fracture has been questioned^12,13^.

Shot peening (SP) is a mechanical treatment applied to the surface of metals to introduce residual compressive stresses that increase resistance to fatigue fracture^14,15^. It is based on the fact that the impact of perfect spheres on a surface produces a superficial plastic deformation and generates stresses. Although the layers below the surface are also affected by the stresses produced, they do not reach plastic deformation and therefore try to return to their original state due to their elasticity; however, the process of returning to their original state is impeded by the plastic deformation of the outer layer and, as a consequence, the underlying layers remain in a stressed state and the residual compressive stresses are finally generated both on the surface and slightly inside the material^15,16^.

Its use has been reported after machining processes or after heat treatments in order to reduce the possibility of the cracks resulting from these processes advancing, leading the material to fracture failure^17^. At the industrial level, SP has been used to increase fatigue resistance in the aerospace industry^18^, as well as in engine components like connecting rods and crankshafts^19,20^; at the medical and dental level, it has been used to increase fatigue fracture resistance in titanium implants^21^ and in dental prosthesis cast clasps^22^.

The use of SP in files made of conventional NiTi has not been reported in the literature; therefore, the objective of this article is to evaluate the resistance to fatigue fracture of manual files made of conventional NiTi after being subjected to the SP procedure. The null hypothesis was that there is no difference in fatigue resistance between files submitted to SP and those not submitted to SP.

## Materials and methods

The sample size was calculated based on a comparative study of the application of thermal treatments on the resistance to fatigue fracture in endodontic files^23^. The software used was PS (Power and Sample Size Calculation Software Version 3.1.2, Vanderbilt University, Nashville, TN, USA), and the number of cycles to fracture (NCF) was used as the result. Considering an alpha (*α*) value of 0.05 and a beta (*β*) value of 0.2, that is, a power of 80% and a level of significance of 5%, the estimated sample size was 20 files per group.

Forty conventional NiTi manual files NITIFLEX (Dentsply Maillefer, Ballaigues, Switzerland), number 30 (that is, a diameter at the tip of 0.3 mm, with a taper continue of 0.02 mm each mm, and a length of 25 mm) were used. They were divided into two groups (n = 20). Group 1 was the control group without SP, and group 2 was composed of files submitted to SP.

### Shot peening procedure

**Figure 1:**
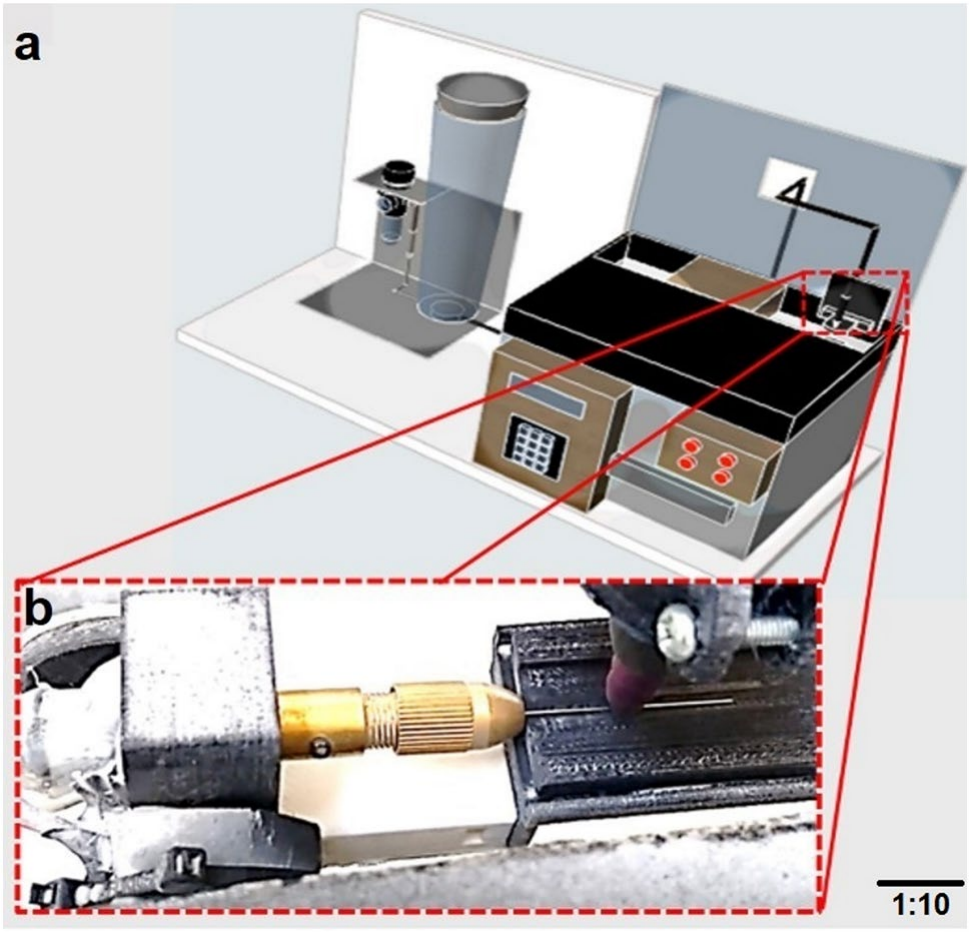
Device for applying the shot peening procedure. (**a**) Schematic representation of the shot peening device. (**b**) Magnified detailed view of the elements directly involved in the shot peening procedure.

**Figure 2:**
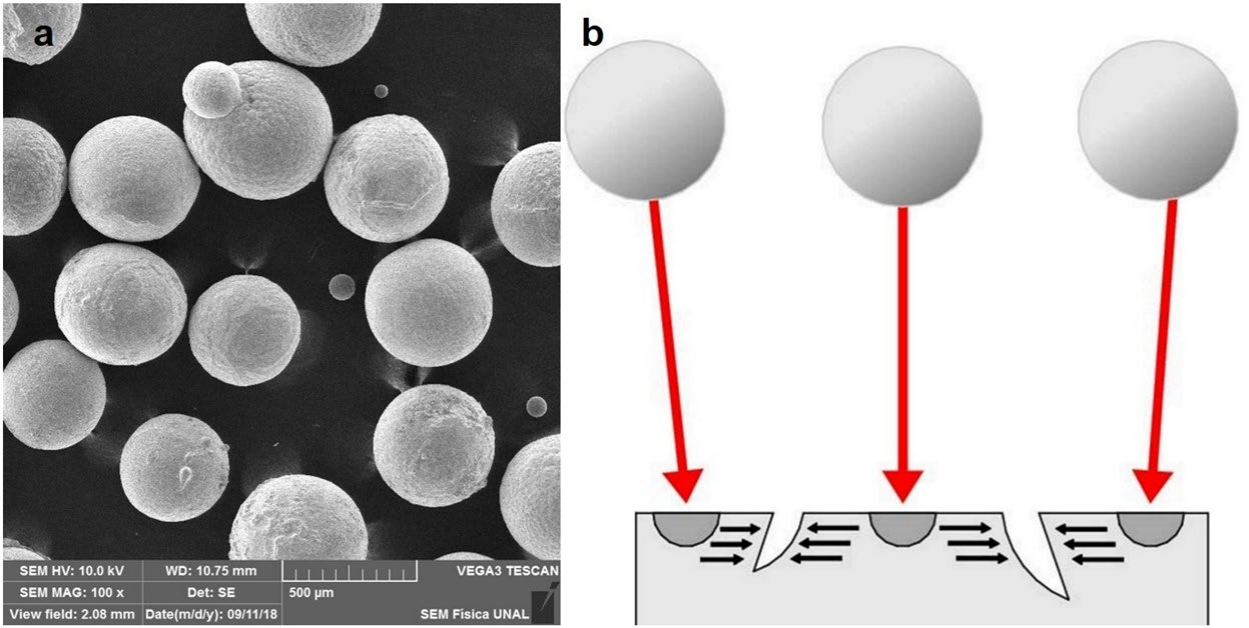
(**a**) S70 grit steel spheres used for the shot peening procedure by SEM (× 100). (**b**) Representation of the effect of shot peening showing how the impact of the spheres on the material surface induces crack closure.

A prototype device (Fig. 1a) consisting of a precision fine sandblasting unit with a pressure gauge (BASIC MOBIL, Renfert GmbH, Hilzingen, Germany) and a connection to an air compressor (INGERSOLL RAND, Wisconsin, USA) was designed and constructed for the SP application. The sandblasting unit was mounted on a wooden base and its air hose connected to a box made by 3D rapid prototyping (PRUSA I3, Prague, Czech Republic) in polylactic acid (PLA) (NATURE WORKS, Minnetonka, USA). Inside, the end of the hose was connected to a Bronze IT Nozzle Tip with a hole of 2.0 mm diameter (RENFERT GMBH, Hilzingen, Germany) (Fig. 1b). The SP media consisted of S70 grit steel spheres (Wujiang District, Suzhou, China). To confirm the spherical shape of the peening media, images by SEM was obtained at 100X (Fig. 2a). A Nema-17 step by step motor (EVA ROBOTICS, Queensland, Australia) that rotates at 1.8° per pulse with a torque of 3 kg was also inside the box, which had the function of displacing the platform where the file lies, back and forth. The box also contained a reduction motor (ARDOBOT, Medellin, Colombia), which rotates the file at 100 rpm while the SP is applied. Both motors were controlled by an Arduino microcontroller board (ARDUINO MEGA 2560, Ivrea, Italy) with a driver (POLOLU, Las Vegas, Nevada) that allowed the turning movement and the displacement of the file to be controlled. A mini adapter (Drill Bit Collet Mini Twist Tool Chuck Set, Koyot, Shanghai, China) was fitted to the shaft of the reduction motor where the active parts of the files were placed after the handle was removed.

Based on pilot tests carried out to define the parameters that would make it possible to achieve adequate coverage and intensity of SP, the following constant parameters were determined: a distance of 3 mm between the tip of the nozzle and the surface of the file, and a 90° angle between the surface and the nozzle.

The intensity of SP was obtained with Almen strips type N, grade I-S (ELECTRONICS INC., Mishawaka, USA), observing an arc height of 0.20 mm (8 N) with a pressure of 0.15 MPa (1.5 bars).

**Figure 3:**
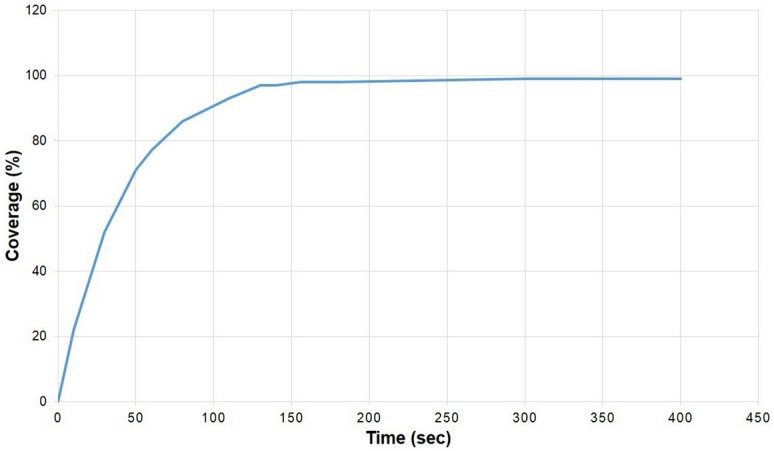
Coverage (%) versus time (s) curve of the application of the SP procedure in endodontic files.

The coverage (*C*) of SP was calculated and applied in agreement with the Avrami equation^24^ Eq. (1). The device displaced the file backward and forwards, while the hose applied SP when the file rotated 30° clockwise. The device repeated the SP procedure until the process completed one full turn; the complete process on the endodontic file took 156 s to obtain approximately 98% coverage (Fig. 3). The coverage could also be seen and verified through observation with a focal variation microscope (FVM).

1 $$ C = 100\% ~X~1 - \left( {e^{{ - \pi r^{2} Rt}} } \right) $$

where r is the radius of peening media, R is the uniform rate of creation of impressions, and t is the time in seconds.

A part of the SP process can be seen in Supplementary video S1.

### Fatigue fracture assessment

**Figure 4:**
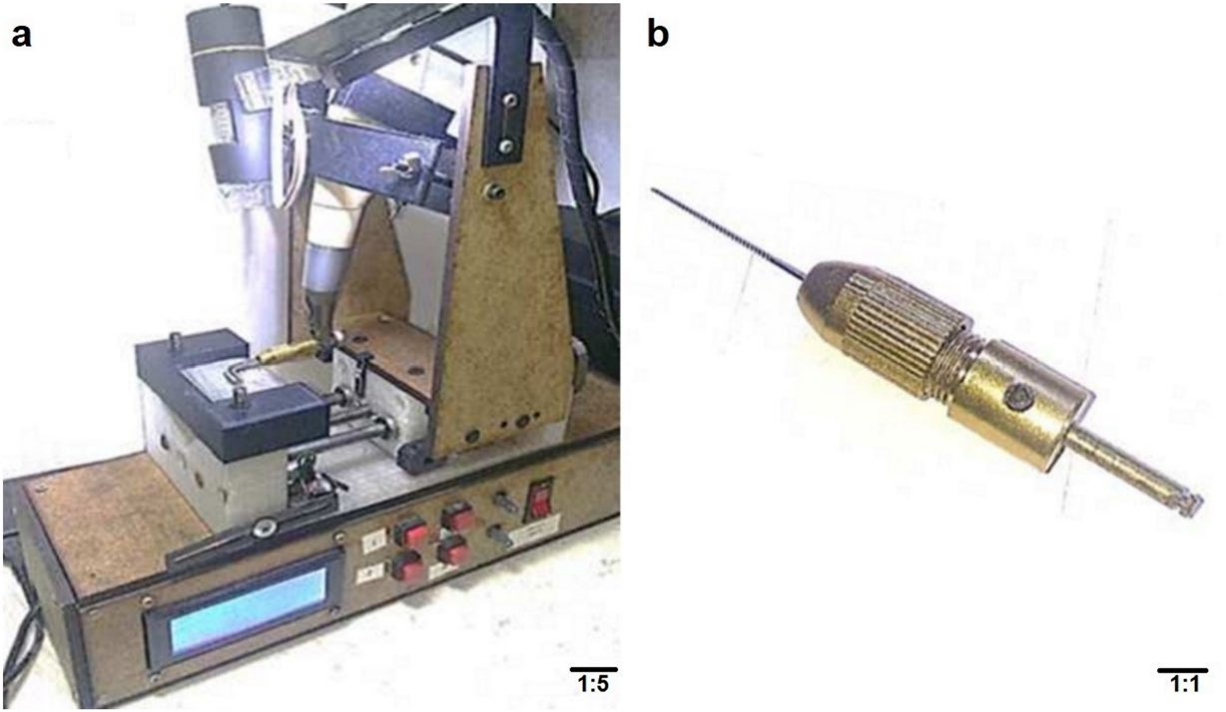
Device for performing the fatigue fracture test. (**a**) Fatigue device. (**b**) Mini Twist Drill Tool Chuck with machined adjustment to fit the endodontic motor.

The fatigue fracture test was carried out with a device built for this purpose (Fig. 4a), which included a simulated stainless steel canal with a curvature of 86° and a total length of 21 mm, where the arc measured 9 mm, the longest straight segment was 8 mm, the shortest straight segment was 4 mm, with a diameter of 1.5 mm and a curvature radius of 6 mm, in agreement with the procedure presented by Rodrigues et al. and Lopes et al.^25,26^. The files were placed in a mini twist drill tool chuck (Electric Drill Bit Collet Mini Twist Drill Tool Chuck Set Pretty RF, Koyot, Shanghai, China), into which a cooper shaft was introduced. The free end of the shaft was machined to obtain the same shape as the setting of the handle of the rotary files that fit in an endodontic motor (Fig. 4b). The active parts of the hand files were then secured in the mini-adapter, as for the SP procedure. Once in position, they were operated with a rotary endodontic motor TRIAUTO ZX (J. Morita Corp., Kyoto, Japan) at 280 rpm and torque 2.5 Ncm, in continuous rotation until fatigue fracture occurred as a consequence of the tension and compression stresses generated alternatively on the analyzed instruments. In all groups, synthetic lubricant (WD-40 COMPANY, Milton Keynes, England) was used to decrease friction between the files and the simulated canal. The fatigue fracture time was measured with a digital stopwatch (THOMAS SCIENTIFIC, Swedesboro, New Jersey, USA) and corroborated by the observation of a video made with a digital camera (AMSCOPE, Irvine CA, USA), which was positioned at the top of the fatigue device. The NCF of each instrument was obtained by multiplying the fracture time in seconds by the rotation speed of the motor (280 rpm) and dividing the result by 60 s. The measurement of the fractured fragment was carried out by means of a digital caliper (DIGIMESS, Sao Paulo, SP, Brazil). The SP device plane and the cyclic fatigue device plane can be seen in Supplementary plane S1.

### Determination of residual compression stresses by X-ray diffraction (XRD)

A file was taken at random from each group and subjected to a residual stress analysis by XRD, which was performed with a Panalytical Empyrean diffractometer (MALVERN PANALYTICAL, Malvern, UK). The measurement interval (°2*α*) was between 47.331° and 51.469°. Using a cobalt anode, two spectral lines were taken into account: Co *kα*_*1*_, with a value of 1.789 (Å), and Co kα_2_, with a value of 1.793 (Å). The ratio of *kα*_*2*_*/kα*_*1*_ was 0.500, and the step size was 0.197°. For the identification of phases, the program X’Pert HighScore Plus 2.2 (MALVERN PANALYTICAL, Malvern, UK) was employed, using the Powder Diffraction File database PDF-2-2004 of the International Center for Diffraction Data (ICDD).

### Surface finish/roughness

To conduct a comprehensive roughness analysis, we took three-dimensional measurements into account to analyze the topography of the material throughout its area, using Sa area parameters. This analysis was performed using an Alicona infinite focus G5 FVM (IFM, ALICONA IMAGING, Grambach, Graz, Austria) in a randomly chosen file. We adjusted each file's handle to a drill chuck (ALICONA IMAGING, Grambach, Graz, Austria) adapted to the microscope. Next, we mounted each file on the 3D rotation unit; the file was positioned and focused, taking a value of 0 for the coordinates of the X, Y, and Z axes; the file was automatically moved towards the lens at a distance of − 3.5 mm on the X-axis, to locate it in the respective point measured at 50 ×, and we later defined the measurement window of 324.561 μm * 324.561 μm. The measurement of the Sa parameters was carried out using the Surface Texture Measurement module of MeX 6.1 software (ALICONA IMAGING, Grambach, Graz, Austria).

### SEM

Again, a file was randomly chosen from each group and was cleaned by immersion in an acetone bath for 2 min. Its fractured surface was then observed, and an EDX analysis was performed with a TESCAN VEGA 3 SEM (Libušina tř, Brno-Kohoutovice, Czech Republic) and EDX BRUKER QUANTA X MICROSOUND (Bruker, Berlin, Germany) in order to observe the fracture characteristics at 500 × and elemental composition of the NITIFLEX files. Images of the surface of the files were also taken at 200 ×, before and after being subjected to SP.

### Statistical analysis

The data were initially analyzed using the Shapiro–Wilk test, in order to confirm a normal distribution; a t-test was then performed to analyze the fracture time, NCF, and length of fractured fragments. The level of statistical significance was 5%, and the software used was STATA 12 (StataCorp, Texas, USA).

## Results

The Shapiro–Wilk test indicated that the distribution of the data was normal (*p* = 0.4383). The results of the time to fracture, the fatigue fracture test (NCF), and the length of the fractured fragments are presented in Table 1. With regard to time to fracture and NCF, statistically significant differences were observed between the two groups, with the highest fatigue resistance being found in the group that was treated with SP (*p* ≤ 0.001).

**Table 1:** Comparison of Nitiflex files with and without shot peening (SP), in terms of the amount of time to fracture (s), number of cycles to fatigue (NCF), and the length of fractured fragments. The surface strain amplitude (%) for one sample per group and the uncertainty type A of each of the results were also calculated.

Groups	n	Time to fracture (s) Mean ± SD	NCF Mean ± SD	Fragment length (mm) Mean ± SD	Surface strain amplitude (%)
Nitiflex file without SP	20	174.2 ± 47.2	813 ± 220	5.35 ± 1.07	11
*Uncertainty type A*	20	10.55 (6%)	49.19 (6%)	0.23 (4.2%)	N/A
Nitiflex file with SP	20	585.5 ± 177.5	2732 ± 828	5.03 ± 1.28	6
*Uncertainty type A*	20	39.69 (6.7%)	185.15 (6.7%)	0.28 (5.5%)	N/A
*p* value		0.001*	0.001*	0.6157	N/A

*Statistically significant at *p* < 0.05.

It was also possible to calculate the surface strain amplitude for one sample per group using Eq. (2)^27^:

2 $$ \varepsilon  = \frac{d}{{2Rc}} $$

where d is the ratio of the diameter of the fracture cross-section and Rc is the curvature radius. Results are shown in Table 1.

The parameters used in this article allow us to calculate that the endodontic file makes 4.7 revolutions in one second, with alternation of tension and compression stresses directly related to the angle of curvature of 86°.

In the supplementary material, it is possible to see a sample of the cyclic fatigue test of the files that have not been subjected to SP (Supplementary video S2) versus those that have been subjected to SP (Supplementary video S3). There was no significant difference in the length of the fractured fragments (*p* = 0.6157).

Equation (3) was used to evaluate the uncertainty type A of the results obtained^28^:

3 $$ U = ~\frac{{Ds}}{{\sqrt n }} $$

where Ds is the standard deviation and n is the sample size of the experiment. The uncertainty type A results are presented in Table 1.

**Figure 5:**
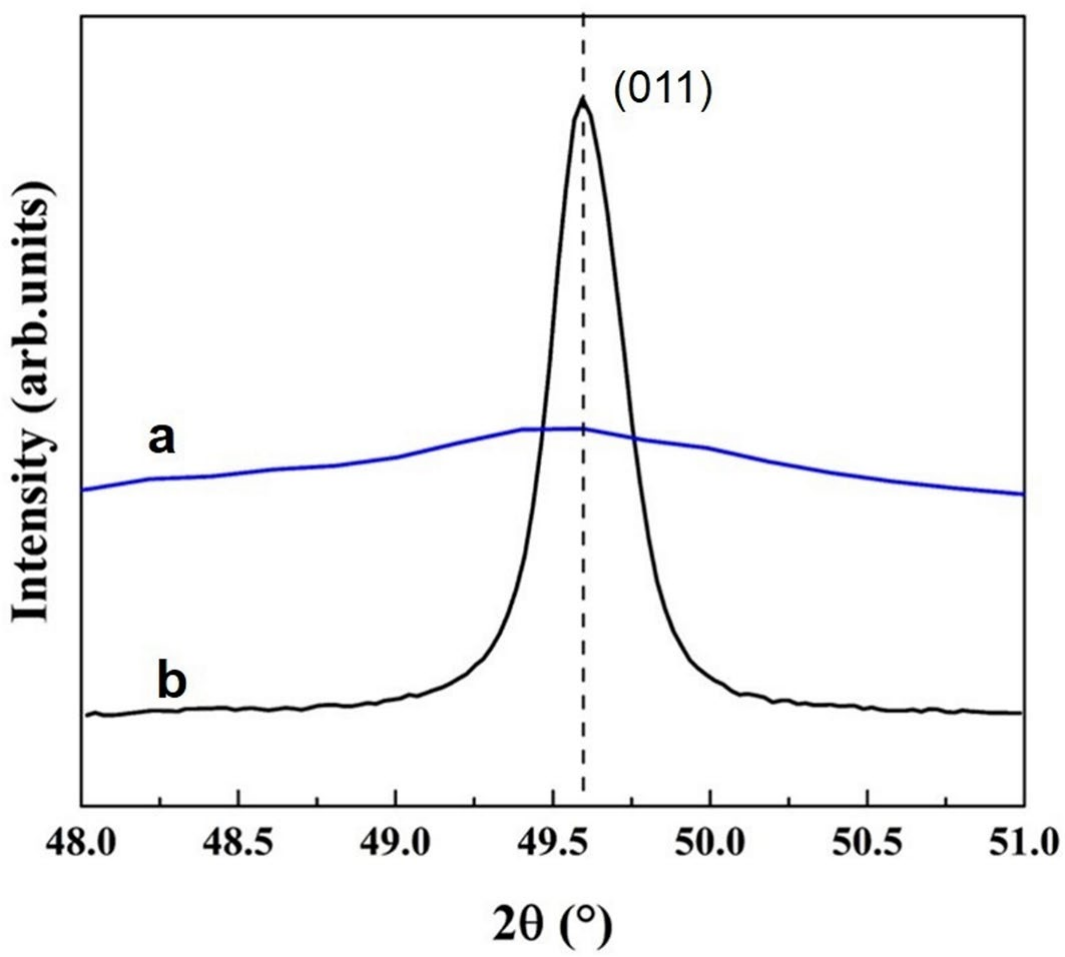
X-ray diffraction pattern. The increase in the width and decrease in the in-plane peak height (011) are shown for the group subjected to the shot peening procedure (**a**), compared to the group not subjected to the shot peening procedure (**b**).

The results of XRD can be seen in Table 2 and Fig. 5. In general, a NiTi alloy with a cubic *β* phase crystallographic structure was observed (PDF card 03-65-4572, ICDD). Interplanar spacing ($d_{{hkl}}$) values were obtained using Bragg’s equation^29,30^ Eq. (4).

4 $$ n\lambda  = 2d_{{hkl}} \sin \theta $$

where n is an integer, λ the anode wavelength, and θ is the measured angle, in which it was possible to calculate a decrease in the interplanar spacing.

**Table 2:** Data calculated in the diffraction plane (011) and the error estimation values derivated from diffractometer: peak position, interplanar spacing (d), full-width-at-half-maximum (FWHM), microstrains (ε), crystal size (D), lattice parameter (a) and stress analysis.

Groups	Nitiflex file without SP	Nitiflex file with SP
Peak position (°2θ)	49.594 ± 0.05	49.597 ± 0.05
d_hkl_ (Å)	2.1328 ± 1.07 × 10^–3^	2.1326 ± 1.07 × 10^–3^
FWHM (°)	0.2892 ± 0.004	1.5882 ± 0.023
Microstrains (ε)	0.1565 ± 0.0002	0.8593 ± 0.001
Crystal size D (nm)	37.30 ± 0.04	6.69 ± 0.007
Cubic lattice parameter a (Å)	9.098 ± 0.01	9.097 ± 0.01
Stress analysis (MPa)	− 144.1 ± 3.82	− 1355.5 ± 47.44

Moreover, an increase in the full-width-at-half-maximum (FWHM) was observed in the file that was subjected to SP compared with the data of the control file. It was also possible to see an increase in the width, as well as the displacement, and a decrease in the height of the peak in the plane (011) in the file that was subjected to SP compared with the control file. Micro strains (ε) were calculated using Eq. (5)^31^:

5 $$ FWHM = 4\varepsilon \tan \theta $$

where a correlation between the line profile, microstrains, and peak position was established. It was seen that an increase in microstrains was also obtained. The crystal size (D) was computed using the Scherrer equation^29,30^ Eq. (6):

6 $$ D = \frac{{K\lambda }}{{\left( {FWHM} \right) \cdot cos\theta }} $$

where K is a constant whose value depends on the shape of the particles (0.89 in cubic systems). The width of the Bragg peak is the sum of the dependent widths of both the diffractometer (FWHM instr) and the measured sample (FWHM s). A standard material (Si) diffraction pattern was taken to determine the instrumental contribution and separate these contributions. The FWHM corrected width corresponding to the diffraction peaks of the samples was calculated using Eq. (7)^32,33^:

7 $$ FWHM = \left( {\left( {FWHM_{s} } \right)^{2}  - \left( {FWHM_{{instr}} } \right)^{2} } \right)^{{1/2}} $$

D values decreased with SP treatment. For a cubic system, the lattice parameter was computed using Eq. (8)^34^:

8 $$ \frac{1}{{d_{{hkl}}^{2} }} = \frac{{\left( {h^{2}  + k^{2}  + l^{2} } \right)}}{{a^{2} }} $$

For an (hkl) system, cubic lattice parameter a was obtained with the latter equation. Lattice parameters had a slight change when its comparing the two samples studied.

Stress analysis was carried out through the sin^2^Ψ method^35,36^ Eq. (9):

9 $$ \sigma _{\psi }  = \frac{{d_{\psi }  - d_{0} }}{{d_{0} }}\frac{E}{{1 + \nu }}\frac{1}{{\sin ^{2} \psi }} $$

The family of crystallographic planes was {110}, the Psi angles number used were 0.00° and 10.01°, the elastic modulus (*E*) used was 31,650 MPa, and Poisson's coefficient (ν) was 0.3^37^.

The values obtained indicated that the SP treatment favored compression stress. All these findings confirmed the detection of residual compression stresses in the file submitted to the SP procedure.

**Figure 6:**
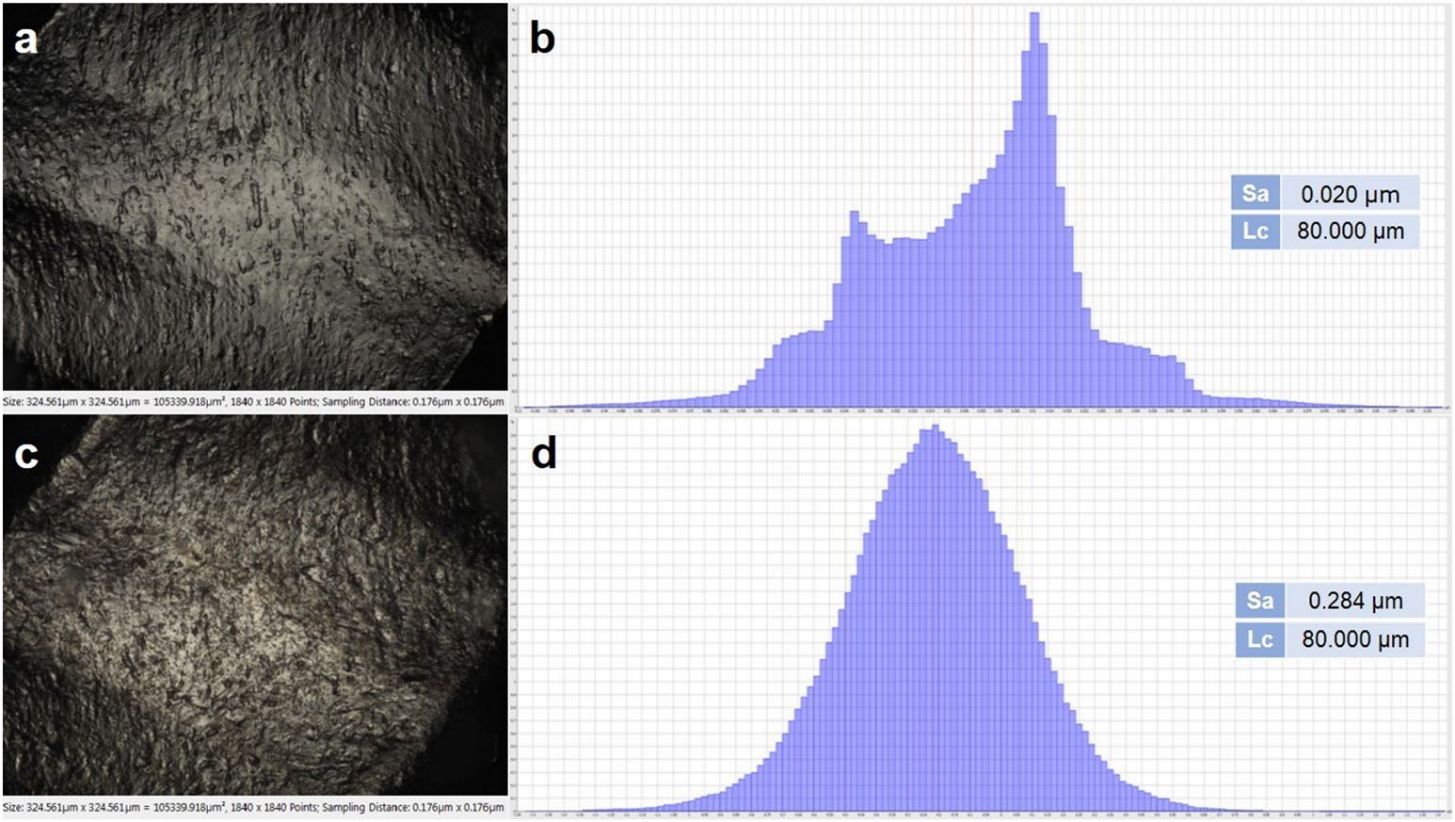
Topographic images taken with focal variation microscope (left) and the respective histogram (right), for the analyzed groups: (**a**) Surface of the NITIFLEX file without shot peening and the corresponding histogram with a deterministic distribution of roughness (**b**). (**c**) Surface of the NITIFLEX file with shot peening and the corresponding histogram with a random normal distribution of roughness (**d**).

The surface roughness by FVM showed a Sa value of 0.020 μm for the file without SP (Fig. 6a). The surface roughness increased to 0.284 μm after the file was subjected to SP, with obvious plastic superficial deformation present (Fig. 6c). The histogram presents a deterministic roughness in the file before SP (Fig. 6b), possibly due to the machining process by which it was manufactured. It is very interesting to note that a random normal distribution of the roughness can be seen as a consequence of SP in the histogram of the sample subjected to SP (Fig. 6d).

**Figure 7:**
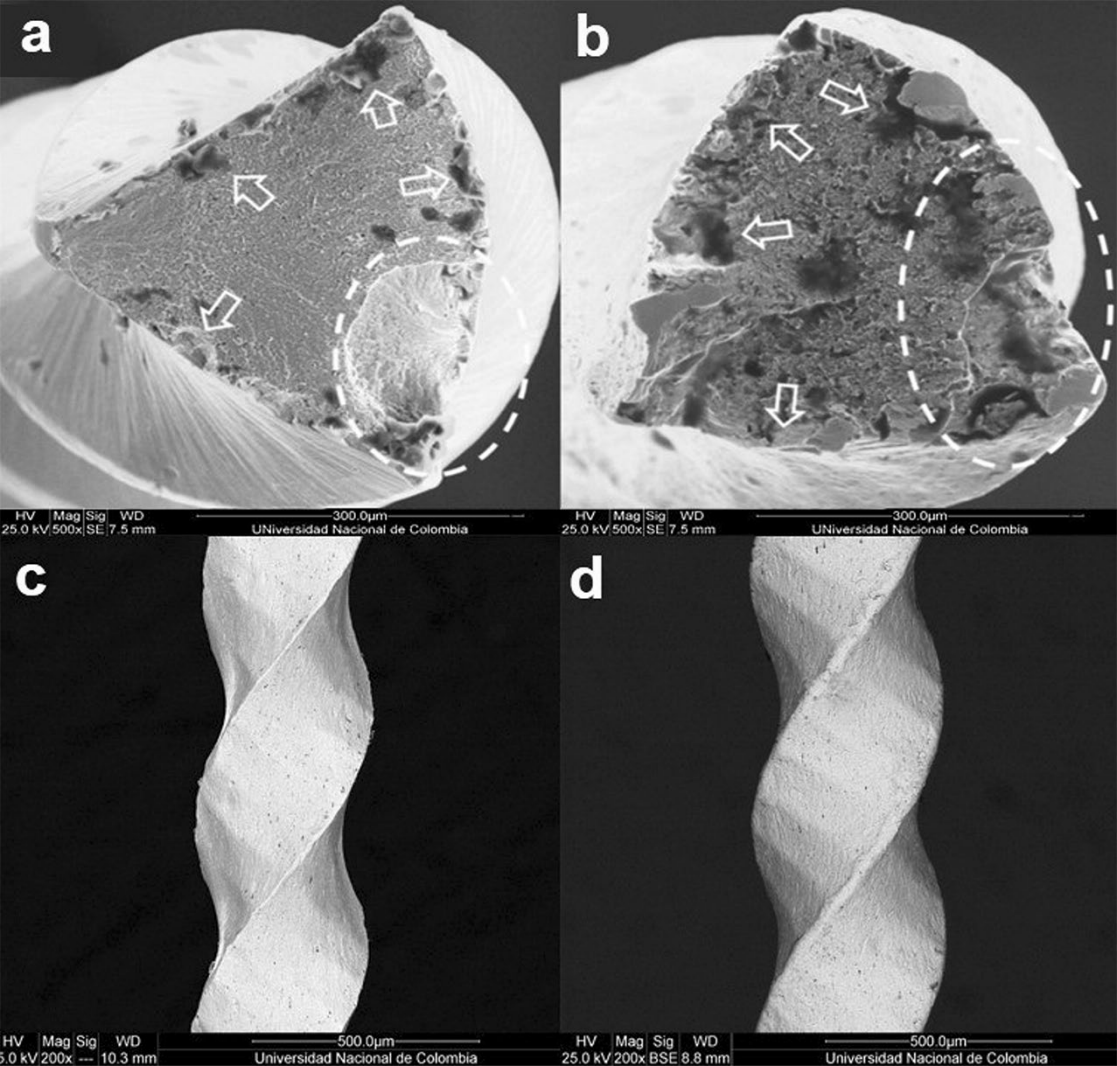
Scanning electron micrographs of the analyzed groups: (**a**) Fractured surface of the NITIFLEX file without shot peening (× 500). (**b**) Fractured surface of the NITIFLEX file with shot peening (× 500). The images show ductile fractures with origins of the cracks (white arrows), final abrupt breakage (circled area), plastic deformation, and central fibrous areas with the presence of dimples. The lateral surface of the NITIFLEX file without shot peening (× 200) (**c**), shows less superficial plastic deformation, compared to the NITIFLEX file with shot peening (× 200) (**d**).

**Figure 8:**
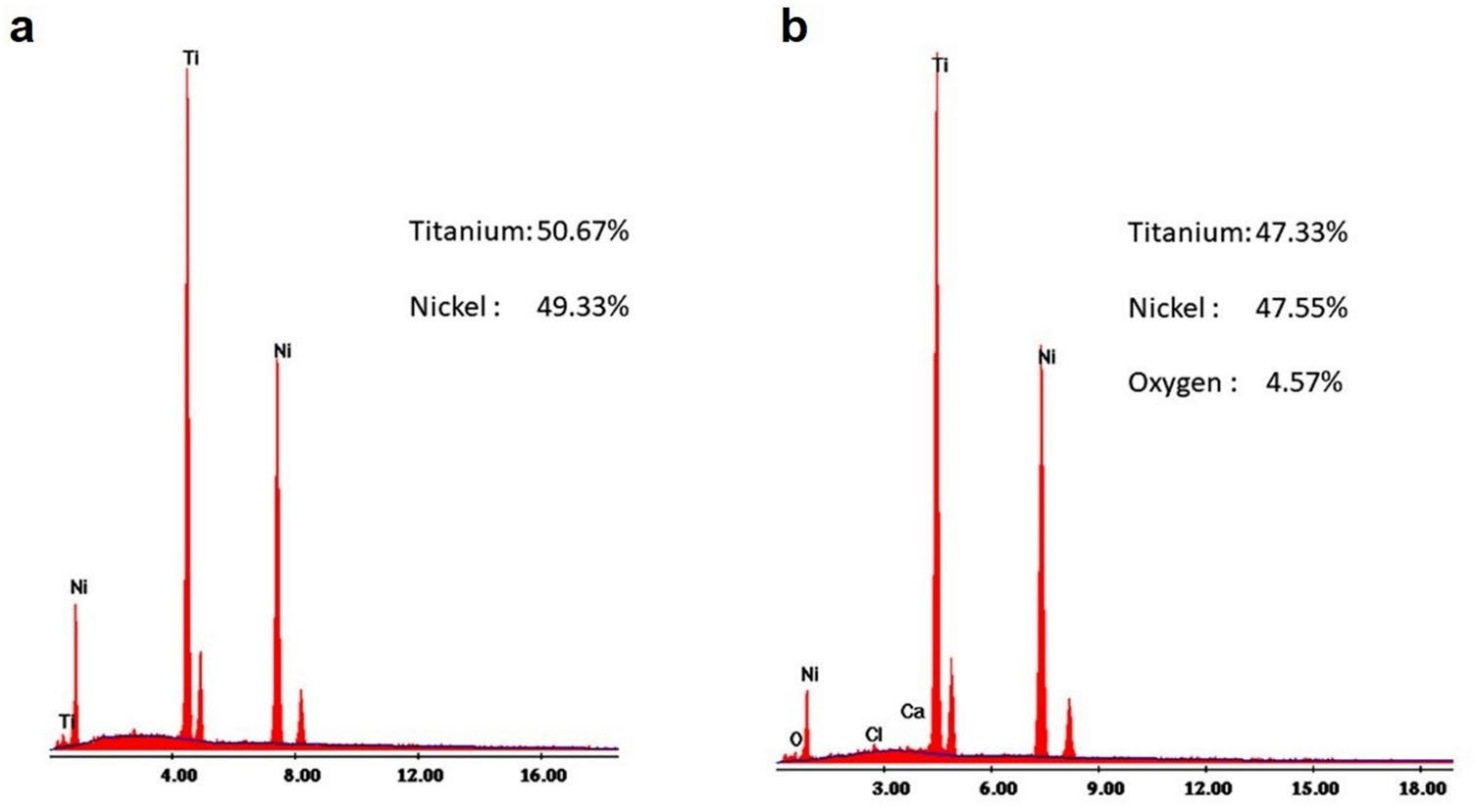
Energy dispersive X-ray spectrophotometric analysis of the composition of a NITIFLEX file: (**a**) Before shot peening. (**b**) After shot peening. In both cases, an almost equiatomic ratio can be seen between the nickel and titanium.

After fractographic analysis of the surface, the SEM observations revealed a ductile fracture with sites of crack initiation, breakage areas, and central fibrous zones, as well as zones of fatigue failure, in both cases (Fig. 7a,b). In addition, the presence of greater roughness and surface plastic deformation was visible in the files subjected to SP (Fig. 7d), compared to the files without SP (Fig. 7c). EDX analysis showed an almost equiatomic ratio between nickel and titanium, with a small amount of oxygen in the file submitted to SP (Fig. 8).

## Discussion

In the present research, we found that application of the SP procedure to files made of conventional NiTi improved their resistance to fatigue fracture when compared to conventional NiTi files. Although SP has been reported to improve resistance to fatigue fracture in other areas of dentistry and medicine^22,38–40^, its use in endodontic files has not been reported previously in the literature.

It is important to mention that NITIFLEX manual files are classic instruments used in endodontics. They are manufactured from conventional NiTi alloy, without the presence of previous thermal or surface treatments^10^, and have a simple triangular design, making them suitable for this research. It has been proposed that the proper way to evaluate a surface treatment is to use only the same type of file, with and without the application of the surface treatment^26^. This was taken into account in the present study, where only NITIFLEX files were used in order to eliminate the influence of additional variables such as manufacturing material, transversal design, taper, pitch, and cutting ability, which could influence the results of the fatigue fracture resistance. Thus, the difference in resistance to fatigue fracture should only be related to the SP application.

In this research, we decided to perform the fatigue test at room temperature, taking into account a recent paper exposing that body temperature does not affect the results of the fatigue test^41^. The reported decrease in NCF is not due to the heat that could hypothetically be transferred from the body, but rather to the temperature transferred by the walls of the artificial canals or by warm water, both as a consequence of the in vitro design of the test. It has been postulated that the real temperature at which the instrument is used in the root canal is room temperature^41^. This is justified as being due to the thermal insulating property of the dentin^42^ and the irrigant used^43^, which is generally stored and used at room temperature. For these reasons, studies on the influence of body temperature in cyclic fatigue present ambiguous results^44–46^. In addition, the SP procedure presents good results when performed at room temperature^47^.

The resistance of PATHFILE and SCOUT RACE rotary files (FKG, La Chaux-de-Fonds, Switzerland) made of conventional NiTi alloy to fatigue fracture has been evaluated twice^48,49^, and NCF results were similar to those found in the present study for NITIFLEX files, which are also manufactured from conventional NiTi alloy.

High flexural resistance of NITIFLEX files has been reported previously^50^, which is consistent with our results, as we found that NITIFLEX files have good fatigue fracture resistance, which may be related to good flexibility.

Unlike the heat treatments that have been used on conventional NiTi alloy to improve its resistance to fatigue fracture, SP is one of the mechanical treatments that acts on the surface of the material. These mechanical procedures also include electropolishing. Although both procedures aim to prevent the formation and propagation of cracks during cyclic loading, the mode by which they tackle the problem is different: electropolishing eliminates surface irregularities and stress concentrators by polishing and wear^11,26^, while SP induces residual compressive stresses under a plastic deformation layer, which also aims to eliminate the existing cracks and their propagation (Fig. 2b), as well as the surface defects that can act as stress concentrators under cyclic loads^16,51^.

It should be noted that, unlike residual tensile stresses, which are harmful to the resistance of the material and promote the formation of cracks, if residual compressive stresses are incorporated into the surface of a metal, such as the NiTi of the endodontics files, it is possible to avoid the propagation of a crack that can appear as a product of repeated loading, which will obviously reduce the probability of cyclic fatigue fracture.

The non-invasive method normally used to assess the presence of residual stresses in a material that has been subjected to SP is XRD^52,53^. The effect of SP on stainless steel bars has been evaluated using XRD, with an increase in width and a decrease in intensity of the peak reported in the respective XRD profile of the sample submitted to SP^54^. This may be due to the lattice strain behavior, uniform tensile strain, and compressive strain. These results are similar to those observed in the present study, where we see a similar behavior in the XRD profile of the file submitted to SP.

The FWHM is defined as the amplitude of the peak at the average height of its intensity, and its measurement is given in degrees^55^. A change of the FWHM could be used to predict the surface coverage of the SP process^36^. In the same way, a decrease in peak intensity is associated with an increase in residual compressive stresses on the surface^36^. The aforementioned coincides with that observed in the XRD graph of the present study, where an increase in FWHM is observed in the sample submitted to SP.

The application of SP to a metal affects its FWHM, increasing its value due to the presence of residual compression stresses^55^. This concurs with the results of the present study, where an increased FWHM was found in the file submitted to SP.

A decrease in the interplanar spacing is related to the presence of residual compression stresses^56^, possibly because the crystalline structure of the metal shrinks due to the loading received and the planes perpendicular to the applied loadings thus become closer to each other, with a consequent clear decrease in the interplanar spacing and a variation in the intensity and shape of the peak, as was also observed in the present study.

In the same way, the crystal lattice does not show changes unless it is affected by stresses^35^. Overall, if a crystalline structure is subjected to tensile stresses, the interplanar spacing decreases perpendicular to the direction of the stresses; therefore, the interplanar spacing could be increased parallel to the direction of the tensile stress^35^. These findings are also in line with our results: when the endodontic files were subjected to SP, the interplanar spacing decreased in the direction parallel to the application of SP.

An increase of microstrains has previously been reported in samples submitted to SP^53^, which is consistent with the findings of the present study, where we observed a higher value of microstrains in files submitted to SP.

The surface strain amplitude is an important characteristic in low cycle fatigue tests, as in this case. It is directly associated with the fatigue life of structural components via the Coffin–Manson equation. The results of this study are similar to those reported for Shen et al., who found that the NCF decreased when the surface strain amplitude (%) increased^27^; in our study, we found that 11% of surface strain amplitude correlated with the low fatigue cyclic resistance, while 6% of surface strain amplitude was related with greater fatigue cyclic resistance.

A limitation of this study was that we analyzed only one endodontic file per group; however, we think it is possible to complete this data in other research. Another limitation in the present study is that the complex geometry of endodontic files difficult to measure the stresses produced in our experiment; the only way to calculate these stresses is by using finite element analysis. This becomes a limitation of our study and is an opportunity to carry on a different investigation in the future.

Roughness has been globally evaluated in two parameters: profiles and areas. In the literature, the most widely used parameter is the Ra profile parameter and the most used area parameter is Sa^57^. Sa has been reported to have a better topographic evaluation of the three-dimensional surface than Ra. Additionally, the Ra value will depend on the direction in which the profile is traced, while the Sa value takes the average of the peaks of a determined area; thus, its results have been considered more reliable^57,58^.

The roughness of the samples treated with SP increased by approximately 10 times compared with those that were not treated with SP, which agrees with that previously reported^59^. The roughness analysis also gives an idea of the quantification of the plastic deformation suffered by the material as a consequence of the SP.

The presence of ductile fractures with dimples and fatigue zones in the files made of conventional NiTi alloy is a normal finding^60^ and coincides with the observations made in the present study, in which different zones of crack initiation were also observed. The fracture may have started in these areas; specifically, the catastrophic failure occurred in the areas indicated by the dotted ellipse lines (Fig. 7a,b). It is also worth noting that these two images confirm that the fracture occurred at the cutting angle of the file in both cases. In Fig. 7a, the endodontic file presents the characteristic beach lines of a fatigue failure in the highlighted area; this can infer that it was at that specific point that the failure advanced, until the instrument fractured, despite the different fracture initiation sites indicated by the white arrows in Fig. 7a,b,. Likewise, it could be assumed that the additional crack initiation sites, visible in Fig. 7a, finally contributed to the fracture occurring by decreasing the surface of the remaining material. On the other hand, a fatigue failure zone can also be observed in the cutting angle (Fig. 7b), which led the instrument to catastrophic failure; however, SP and the elastic–plastic deformation near the surface leads to the formation of compressive residual stresses, which could delay the fatigue crack initiation time and change the crack initiation site^61^, as can be seen in Fig. 7b. This causes the SP to provide greater resistance to fracture, making more crack initiations necessary for catastrophic failure to occur and possibly also distributing the stresses on the surface of the material, which also increases its fatigue resistance.

Superficial plastic deformation can be seen in the images of the lateral surface of the files (Fig. 7d), coinciding with that previously reported in the literature, where it is possible to observe the surface effect of SP on the material and the plastic deformation, depending on the intensity of SP^62^.

EDX analysis presents an equiatomic ratio between nickel and titanium in both cases, which is similar to that reported in the literature^63^.

Regarding the length of the fractured fragment, our results are similar to those reported previously^64^, where conventional NiTi alloy rotary files were used in a simulated canal with a 90° angle, and lengths of 5.13 mm for PROFILE (Dentsply Maillefer, Ballaigues, Switzerland), 5.21 mm for MTWO (VDW, Munich, Germany), and 5.30 mm for K3 files (SybronEndo, Orange, CA) were observed, with no significant difference between them. In the present study, we found lengths of 5.03 ± 1.28 mm for files without SP and 5.35 ± 1.07 mm for files with SP, with no significant difference.

In conclusion, the SP surface procedure increases the resistance to fatigue fracture in files manufactured from conventional NiTi alloy. This increase in resistance to fatigue fracture is due to the presence of residual compression stresses, surface work hardening, and surface-modified finishing with plastic deformation in the files submitted to SP. Further studies should be undertaken to look at the effect of SP on rotary files that have been heat-treated or to perform a comparison with rotary files that have been electropolished, and several experiments could be undertaken using different SP parameters to test how the final results are influenced by the best parameter combination in the endodontic rotary files mentioned.

## Supplementary Information

Supplementary Information 1.

Supplementary Information 2.

Supplementary Video 1.

Supplementary Video 2.

Supplementary Video 3.

## Data Availability

The data generated and analyzed during this study are available from the corresponding author upon request.
