# Supplementary material for: Shot peening increases resistance to cyclic fatigue fracture of endodontic files
Source: Sci Rep. 2021 Jun 21;11:12961. doi: 10.1038/s41598-021-92382-x (PMC8217493; doi:10.1038/s41598-021-92382-x)
Supplement: Supplementary file 1 — Supplementary Information 1. [file 41598_2021_92382_MOESM1_ESM.docx]

**Legends Supplementary material.**

**Supplementary plane S1.** The plane of the Cyclic fatigue device (left) is observed with its respective frontal, lateral and superior view and its three-dimensional reconstruction. The shot peening device plane (right) is observed, with its respective frontal, lateral and superior view and its three-dimensional reconstruction.

**Supplementary Video S1.** SP device showing briefly a little sequence in which the sample is submitted to the SP process.

**Supplementary Video S2.** It can be seen cyclic fatigue test was performed on the endodontic file that has not been subjected to the SP process until the instrument fracture occurs.

**Supplementary Video S3.** It can be seen cyclic fatigue test is performed on the endodontic file that has been subjected to the SP process until the instrument fracture occurs.
