# Supplementary figures and images for: Shot peening increases resistance to cyclic fatigue fracture of endodontic files
Source: Sci Rep. 2021 Jun 21;11:12961. doi: 10.1038/s41598-021-92382-x (PMC8217493; doi:10.1038/s41598-021-92382-x)

Cyclic fatigue device

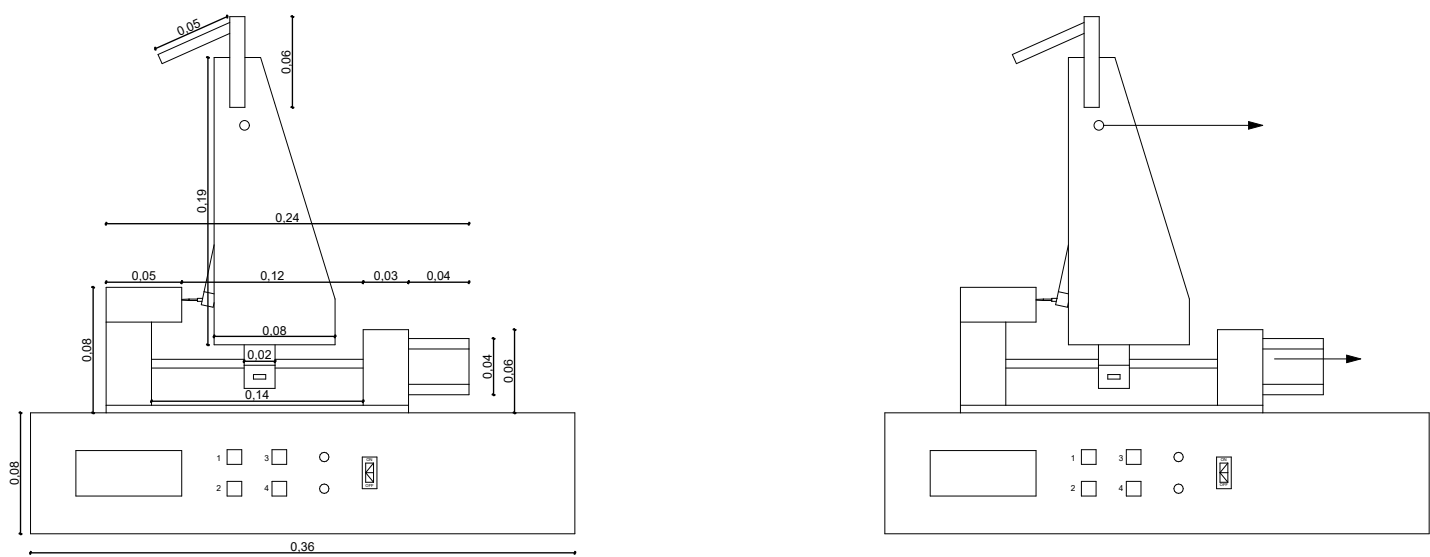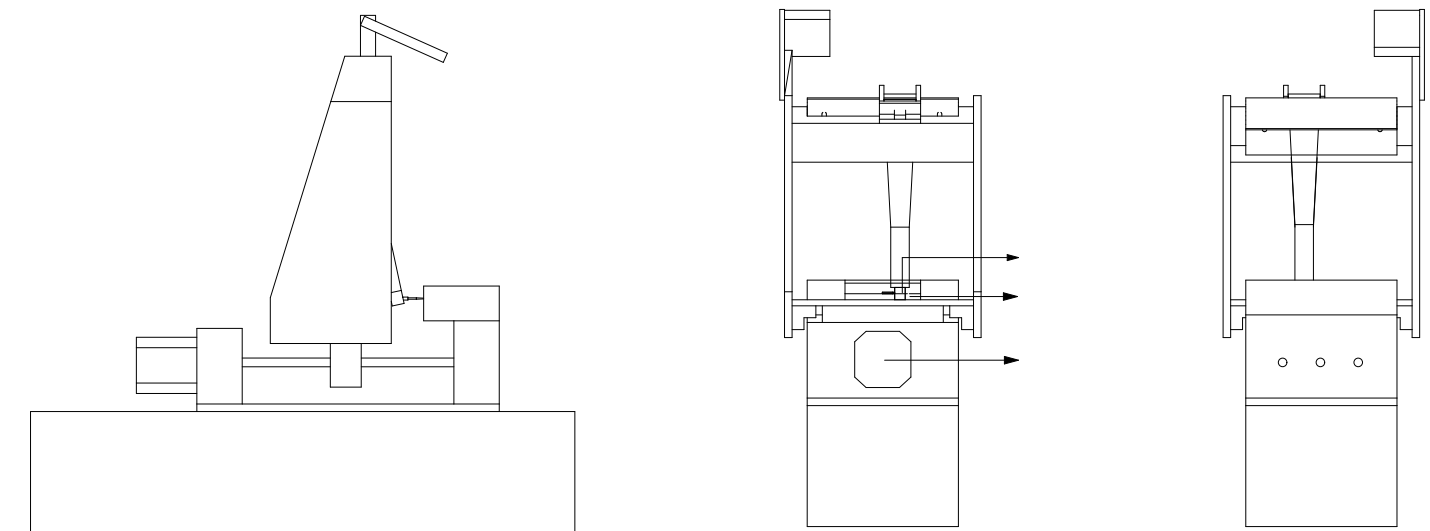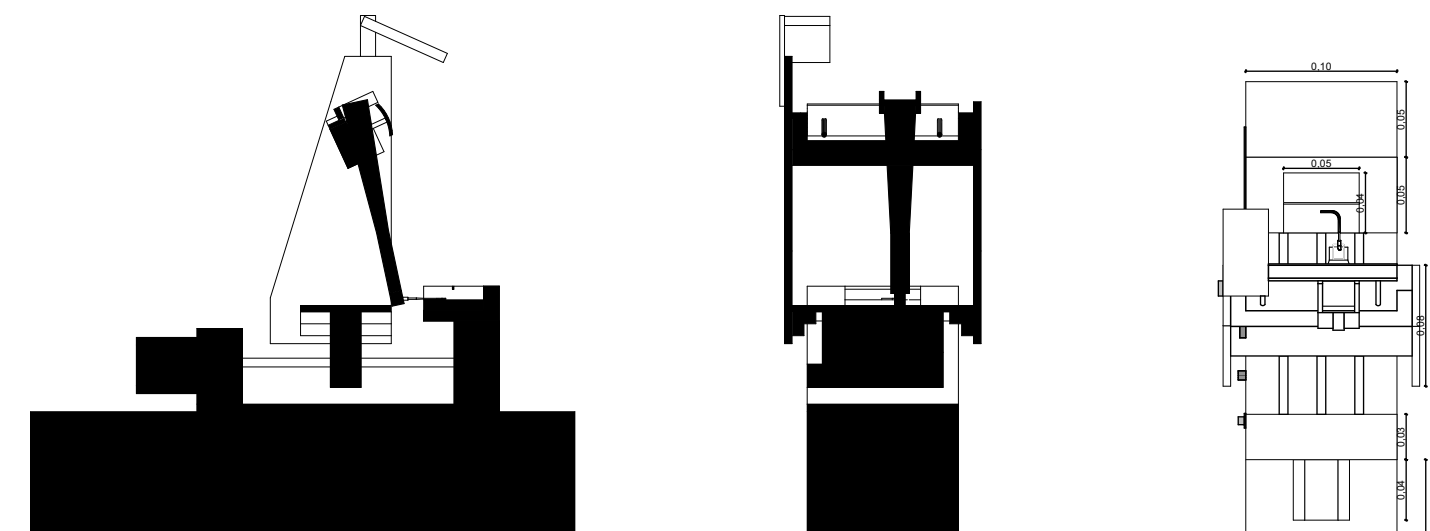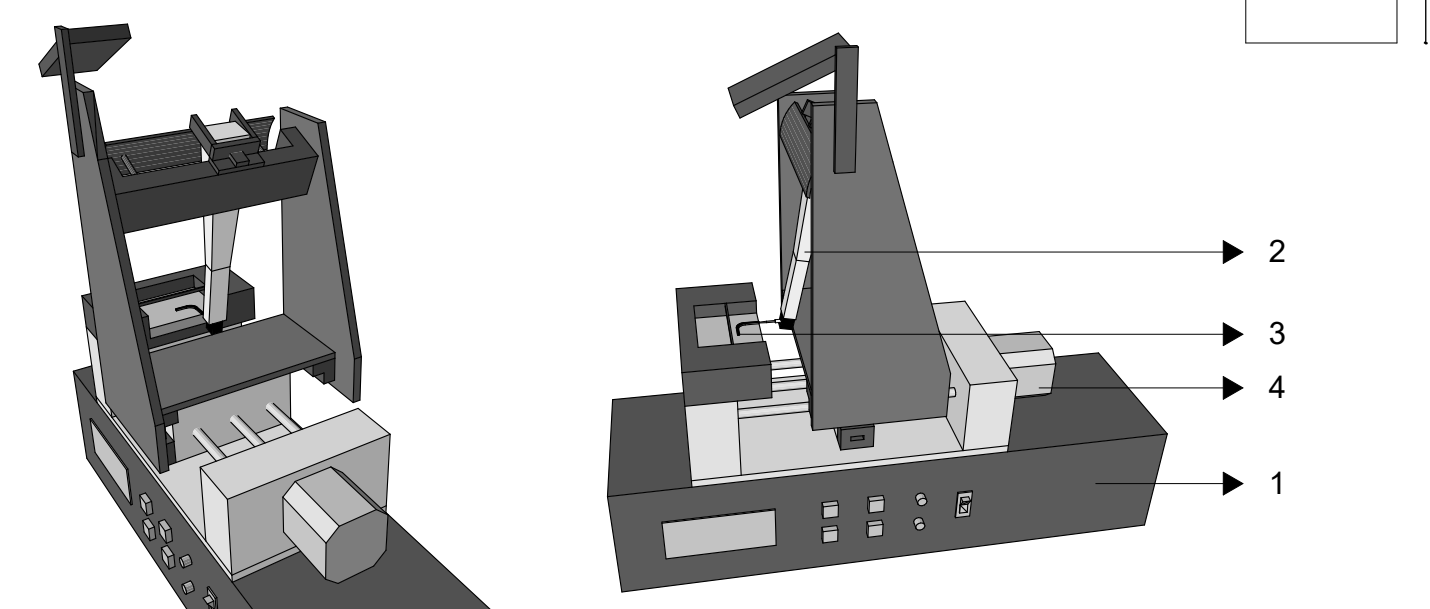

Shot Peening Device

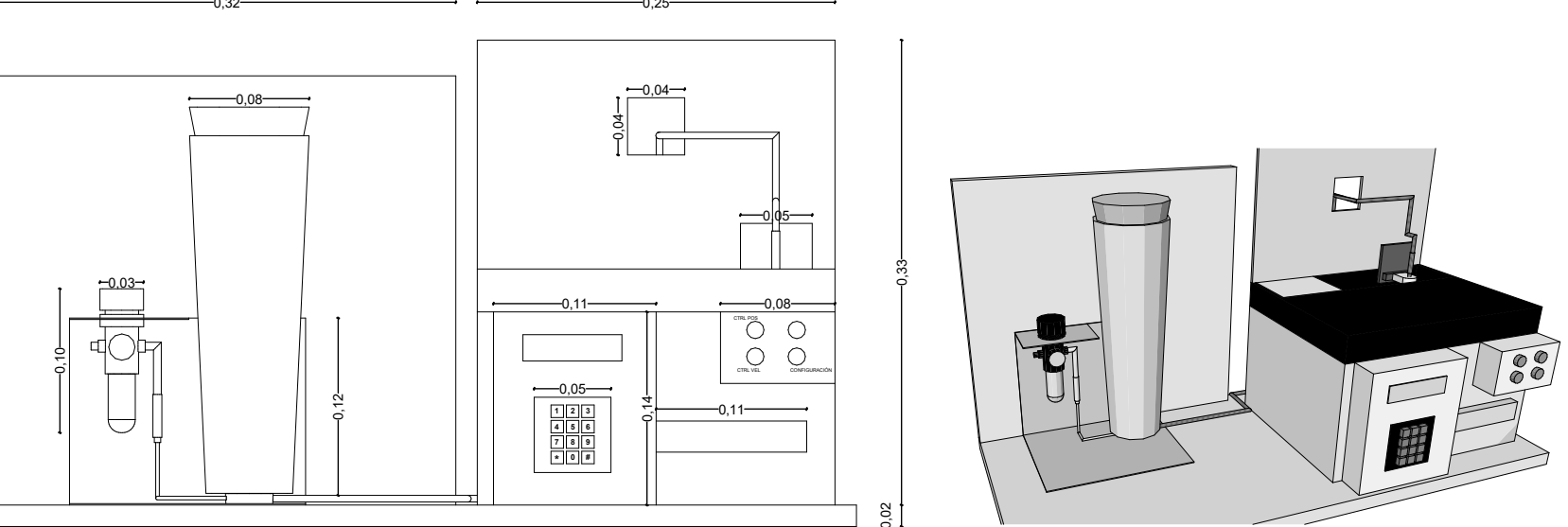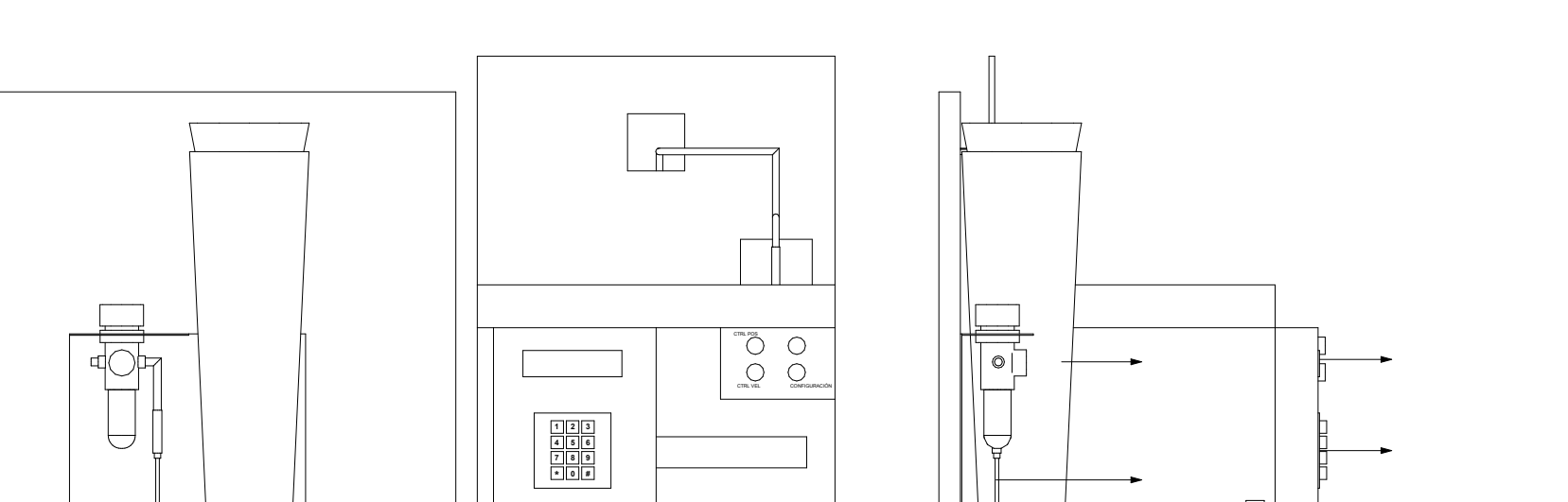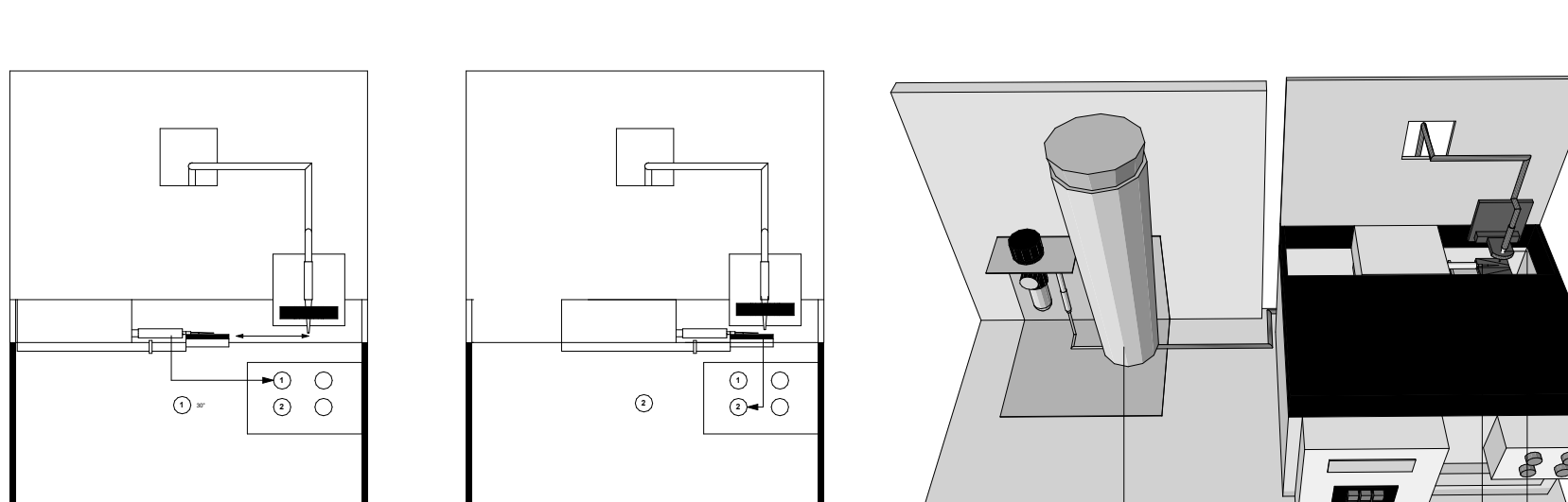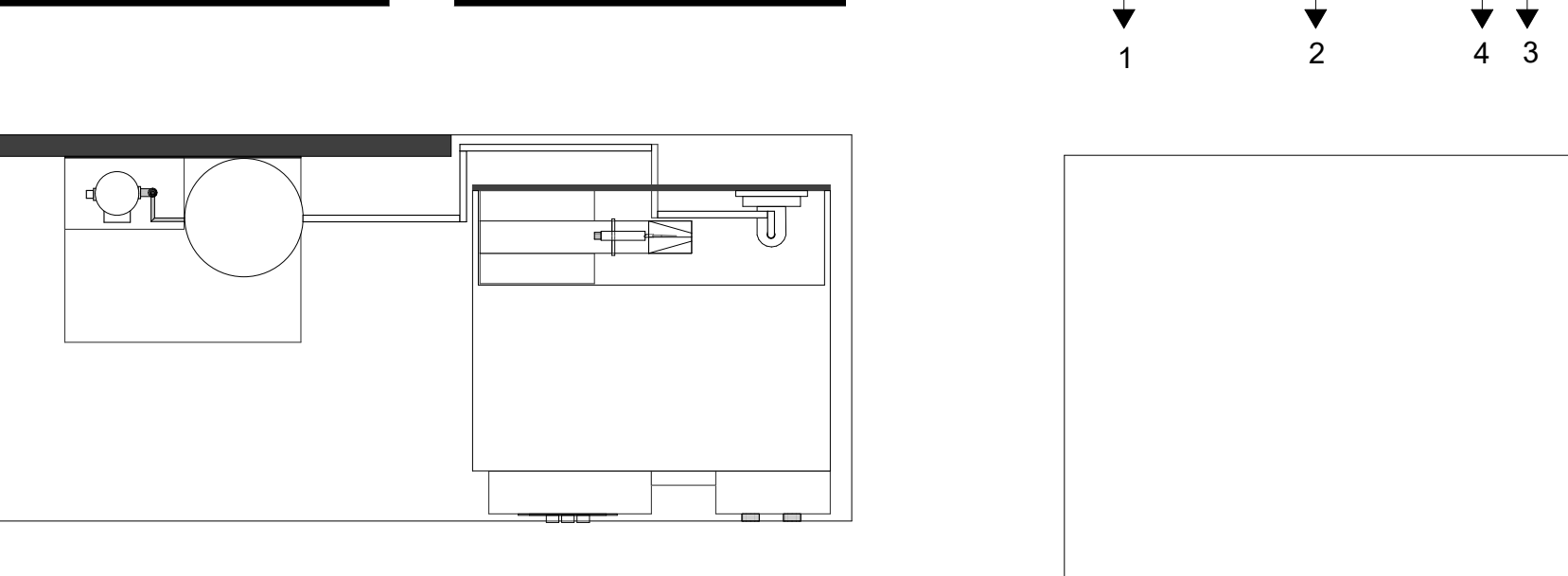

Supplement: Supplementary file 2 — Supplementary Information 2. [file 41598_2021_92382_MOESM2_ESM.pdf]
